# Patient safety culture through the lenses of surgical patients: a qualitative study

**DOI:** 10.1186/s12913-025-12366-9

**Published:** 2025-02-07

**Authors:** Magnhild Vikan, Arvid S. Haugen, Berit T. Valeberg, Ann K. Bjørnnes, Vigdis K. S. Husby, Ellen CT. Deilkås, Stein O. Danielsen

**Affiliations:** 1 Department of Nursing and Health Promotion, Faculty of Health Sciences, Oslo Metropolitan University, St. Olavs Place, P.O. Box 4, 0130 Oslo, Norway; 2 Department of Anesthesia and Intensive Care, Haukeland University Hospital, Bergen, Norway; 3 Department of Orthopedic Surgery, Trondheim University Hospital, Trondheim, Norway; 4 Department of Health Sciences Aalesund, Faculty of Medicine and Health Science, Norwegian University of Science and Technology, Aalesund, Norway; 5 Department of Health Services Research, Akershus University Hospital, Lørenskog, Norway

**Keywords:** Patient engagement, Patient experiences, Patient safety culture, Patient safety, Adverse events, Medical errors, Surgery, Disclosure, Quality improvement

## Abstract

**Background:**

Patient engagement and learning from patients’ experiences may increase patient safety and reduce the occurrence of adverse events. Most adverse events are related to surgery, and patient outcomes are positively associated with patient safety culture. This study aimed to explore former surgical patients’ perspectives and experiences of adverse events and patient safety culture during their surgical pathway and identify themes relevant to adverse event causes and quality improvement projects.

**Methods:**

The design of this qualitative study was explorative, utilizing an abductive approach. We purposefully recruited former surgical patients from Norwegian user organizations based on group characteristics sampling. The participants were 57% men and 43% women, aged 35 to 64 years. We conducted 14 individual semi-structured interviews between 18/01/24 and 07/03/24 using Zoom’s video audio software, with an average duration of 65 min. We analyzed the data using Braun and Clarke’s method for reflexive thematic analysis, and generated themes by examining patterns of meaning throughout the dataset.

**Results:**

Data analysis generated three themes concerning the former surgical patients’ perspectives of patient safety culture and adverse events: (1) “Personalized care and predictable pathways increase patients’ sense of safety”; (2) “Surgical patients’ involvement: Aspire to be a resource – Not a threat”; and (3) “Time to cultivate a culture that fosters improvements and reconciliation.”

**Conclusions:**

This study provided insight into patients’ perspectives on adverse events and patient safety culture in the surgical context. The patients underscored the value of predictable plans in caregiving, tailored information, personalized care, and dialogue on equal terms. They considered the demand for efficiency, professional hierarchy, status, prestige, and authority to be barriers to patient engagement and safety. Interventions to improve a culture of openness, psychological safety, and organizational learning in the surgical context could increase the safety of patients and healthcare professionals. Finally, acknowledgment of adverse events, information, and follow-up were essential for patients and next of kin to move on after an adverse event.

**Supplementary Information:**

The online version contains supplementary material available at 10.1186/s12913-025-12366-9.

## Background

In recent years, patient engagement has been highlighted as essential for improving quality and safety in healthcare delivery [1, 2]. Increased patient participation and patients as co-producers of healthcare systems may contribute to identifying relevant themes for improvement, reducing healthcare costs, and building public trust in healthcare services [2, 3]. Learning from patient experiences is encouraged to improve patient safety at the organizational and national levels [1, 2]. Capturing patients’ experiences through surveys with additional space for comments is valuable and an essential outcome measure of quality [4]. Additionally, listening to, considering, and learning from firsthand experiences can increase clinical effectiveness and improve the impact of quality interventions in healthcare [5, 6]. Thus, patients’ experiences form a pivotal pillar in patient safety and quality research [6].

Twenty-five years after the Institute of Medicine published its landmark report “To err is human,” efforts to reduce the rates of adverse events and enhance patient safety have been continuously prioritized in healthcare systems [1, 6]. The occurrence of adverse events is still too high; it affects 10% of hospitalized patients in high-income countries, and globally, lack of patient safety leads to 3 million deaths annually [7, 8]. Patient adverse events may be described as an omission or commission of actions leading to or having the potential to cause increased risk, patient harm, or injuries related to healthcare delivery and not to the underlying health condition [9]. Of the adverse events, 50% are estimated to be preventable, with the vast majority related to surgery [7, 9]. The adverse events in the complex surgical context are more often severe and necessitate additional treatment; however, there is heterogeneity in the evidence regarding the measurement and monitoring [7, 9]. These injuries entail significant costs; hence, reducing adverse event rates has economic benefits and could increase the total quality of healthcare services [8].

Patient safety in healthcare delivery involves structural and processual activities that sustainably detect risks and reduce preventable harm and the impact of injuries [1, 10]. Responses to an adverse event and communication afterward are essential dimensions of patient safety culture [11]. Patient safety culture refers to the shared attitudes, values, and behaviors toward patient safety at multiple organizational levels, and it encompasses such dimensions as leadership, teamwork, safety climate, and the willingness to learn from adverse events [11, 12]. Previous studies have measured patient safety culture as a proxy for quality of care for the last two decades, most commonly with healthcare professionals responding to questionnaires [11, 13]. Patient safety culture is inversely associated with patient outcomes; thus, increasing patient safety culture is likely to reduce the rates of adverse events [14, 15].

Previous studies have also revealed a discrepancy between healthcare professionals’ and patients’ concepts and perspectives of patient safety [16, 17]. Other studies have demonstrated that healthcare professionals underestimate the burden of adverse patient events [18, 19]. Bishop and Cregan [20] reported that patients and families affected by adverse events felt they were ignored and thought they felt treated more like a number than a person. Harrison et al. [21] reviewed the scientific literature on patients’ experiences of adverse events and reported a lack of in-depth explorative studies of their perspectives. Qualitative methods and in-depth interviews may capture a broader understanding of underlying dimensions within patient safety culture [11, 13].

The crucial role of patient engagement in patient safety efforts and the frequent occurrence of adverse events in the surgical context pointed toward the need for qualitative research on patients’ perspectives. Exploring former surgical patients’ experiences concerning patient safety culture and the theoretical framework of Donabedian’s model of quality in healthcare [10, 11, 13, 22, 23] might enhance understanding of patient safety culture and adverse events. and uncover specific themes of interest for enhancing quality and education within the surgical context. This need for increased insight raises this study's research question: “What are the perspectives of surgical patients on patient safety culture, the potential causes of adverse events, and relevant quality improvement projects in the surgical context?”. Thus, the study’s objectives were to explore the perspectives and experiences of former surgical patients regarding patient safety culture, determine their perceptions of what causes adverse events,

## Methods

### Study design

This qualitative study used an explorative design with an abductive approach to meet its objectives [24, 25]. The overall objective was to enhance the understanding of patient safety culture in surgical care by obtaining patients’ experiences. We interviewed patients with previous experience with surgical treatment to capture their perceptions and perspectives. An abductive approach was appropriate due to the inductive nature of the mind map, interviews, and initial analysis, combined with the deductive approach and inspiration of adding evidence to the theory of underlying cultural dimensions [13, 24, 25]. We used Braun and Clarke`s Thematic Analysis Reporting Guidelines (RTARG) to ensure the quality and reporting of the study [26].

### Participants

We used a group characteristic sampling method and purposefully recruited participants from user organizations in the South-Eastern Norway Regional Health Authority [25]. These organizations aimed to support cancer patients throughout their patient pathways or support patients after adverse events related to healthcare services. User representatives requested pertinent participants based on written information and forwarded the contact data to the first author (MV). We included volunteer former patients with experiences from various surgical specialties. We recruited adult participants who had undergone acute or elective surgery and had no time limit on their experiences. The participants, as intended, represented a strategic sample with heterogeneity regarding age, gender, occupational status, and educational level.

### Data collection

MV conducted 14 interviews between 18/01/24 and 07/03/24. Due to the potentially sensitive information arising from the conversations, we found it appropriate to conduct individual interviews. One week before the interviews, the participants received a mind map to increase their reflections on patient safety and adverse events, recall their experiences, and make the data broader and deeper [see Additional file 1]. We developed a semi-structured interview guide for this study to capture information according to the study’s objectives and align with the mind map [see Additional file 2]. A tertiary hospital user representative participated in formulating the mind map and the interview guide to avoid medical phrases, i.e., the user representative pointed to the need for changing the term ‘surgical patient’ to ‘patient’ and to add ‘injury’ and ‘near-misses incidents’ as more concrete terms than ‘adverse event.’ The content of the interview guide was open and explorative questions due to the main topics in the study’s objectives: Their experience and sense of patient safety from hospital admission until returning home, and their experiences related to adverse events and near-misses incidents. The term patient safety culture was not used in the interview guide, as it might be unfamiliar to the participants. However, when the participants touched on underlying patient safety culture dimensions, MV asked follow-up questions to enrich the data. Due to geographical distances and some participants’ health conditions, the interviews were conducted through a closed meeting in Zoom and recorded in an encrypted and secured storage tool. The audio-taped interviews lasted between 45 and 110 min, with an average duration of 65 min. The audio files were primarily automatically transcribed using the Whisper text tool on a secure server for sensitive data. They were then manually revised by MV [27], resulting in an average of 20 pages / 11,524 transcribed words per interview. Based on the transcripts and audio files, we recruited participants until we achieved sufficient data richness to meet the study`s objectives, following Malterud’s method for assessing information power [28]. MV wrote field notes and unfiltered reflections after each interview to enhance her reflexivity. These reflections were integrated into the discussions to enrich the analysis process.

### Data analysis

The data analysis process followed Braun and Clarke`s method for reflexive thematic analysis [29]. The reflexive thematic analysis consists of six phases and is a reflexive iterative process moving back and forth between the phases. MV used the software Lumivero NVivo 12 and manual visual tools, such as whiteboards, posters, and patches throughout the process. The first phase was familiarization [29]. MV and SOD listened to audio tapes and read the transcripts three times to become familiar with the individual interviews and to consider the dataset as a whole, noting memos and preliminary codes throughout the phase. The second phase was coding, starting inductively and descriptively and staying close to the semantic content of the empirical material. MV reflected on theoretical and deductive approaches and kept these in mind during the second coding. MV redefined the first codes to represent an idea and merged several.

In the third phase, MV and SOD clustered similar codes into candidate themes and generated initial themes. We discussed initial impressions of the dataset and the initial themes to enhance reflexivity and conceptualized the content to interpret the data better. The revised initial themes were assessed toward the raw data to evaluate how they could fit the dataset. During this examination, which was repeated twice, we developed and revised the themes and subthemes representing phase four. MV wrote about how we conceptualized the themes and found descriptive quotations for each.

In phase four, we discussed the analysis process with ECTD and ASH to enhance reflexivity and challenge the interpretations critically. In addition to MV, an operating room nurse and a PhD candidate, this analysis panel consisted of senior researchers with clinical experience: SOD is an operating room nurse, ASH is an anesthesia nurse, and ECTD is a physician. We had a dynamic process moving back and forth between the manifest dataset and the latent and abstracted interpretations. In phase five, MV and SOD discussed, revised, and developed the theme names and revised the additional documents. Each theme represented patterns of meaning and shared understanding throughout the dataset. The iterative process maintained the abductive approach: We searched for data related to the theory of patient safety culture dimensions [11, 13] and themes beyond our preconceptions. To inspire and challenge the dataset’s interpretation, we actively used field notes and were aware of presumptions about our contextual understanding. After feedback from the other authors, MV and SOD made minor revisions, and MV wrote up the results in phase six [29].

## Results

**Fig. 1:**
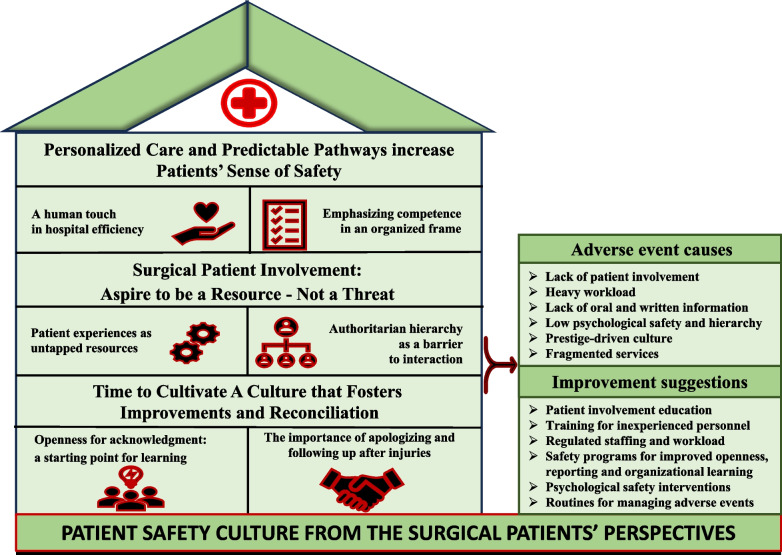
Results presented in three themes, each with two subthemes

The analysis process developed three themes, each consisting of two subthemes. The themes represented patterns in the former surgical patients’ perspectives of patient safety culture and adverse events in surgical pathways. The results are presented in Fig. 1 and detailed in the text and quotations highlighting the themes generated from the dataset. Table 1 presents the demographic characteristics of the 14 participants. The participants had experienced adverse events ranging from lack of information, diagnosis/ treatment delays, and expected complications to severe disabling injuries.

**Table 1:** Demographic characteristics of the participants in the study

Characteristics	Number (n)	Percentage (%)
Total participants	14	100
**Gender**		
Female	6	43
Male	8	57
**Age**		
35–44	3	21
45–54	3	21
55–64	8	57
**Surgical field**		
Orthopedics	4	29
Gastroenterology/gynecology	4	29
Urology	5	36
Other	1	7
**Acute or elective**		
Acute	2	14
Elective	12	86
**Level of education**		
High school	6	43
University	8	57
**Occupation**		
Employed	6	43
Unemployed	8	57
**Years since surgery**		
1–5	8	57
6–10	3	21
≥ 11	3	21

### Theme 1: Personalized care and predictable pathways increase patients’ sense of safety

The participants with previous experience as surgical patients emphasized that the combination of personalized healthcare and a structured pathway promoted predictability, safety, and quality.

#### Subtheme 1a: A human touch in hospital efficiency

The participants highlighted needing personalized care, respect, and friendliness to feel safe during their patient pathway. Healthcare professionals’ qualities promoting patients’ feelings of safety included being proactive, critically observant and solving problems. The former patients appreciated healthcare professionals who were accountable for the patient in the operating room, “*I will take care of you while you are sleeping*” (ID03), and in the surgical ward, *“I am responsible for you this afternoon”* (ID06). They valued a human touch, such as short comments, warm smiles, or *“drawing a smiley face on a piece of paper…to give a little extra that means so much”* (ID02). The participants valued healthcare professionals in intensive care and surgical units who were dedicated and empathic, putting “*their hearts into their jobs*” (ID09).

Nontechnical and communicational skills, such as engaging in respectful dialogue, listening empathetically, and having a humble attitude, enhanced patients` sense of safety. The participants also preferred explicit and unambiguous communication when receiving information. Linguistic barriers, such as the absence of nuanced language, reduced their feeling of safety. Information was perceived as a crucial factor in feeling safe and in control in uncertain situations and having trust in healthcare delivery: “*For me, patient safety means receiving relevant information about what will be done and when it will happen. It reassures me that they are in control of my condition*” (ID08). The participants highlighted the importance of receiving preoperative information about the procedure and its risks directly from the treating surgeon, information through minor procedures, and postoperative information on surgical outcomes. They felt that including caregivers in receiving pre- or postoperative information could alleviate their burden and that providing oral and written information, including postoperative care guidelines and contact details, was critical for safe discharges. A lack of information could undermine a sense of safety: “*I had to see a psychologist because I was scared. I was afraid of dying. I think this fear stemmed from a lack of information*” (ID06).

The healthcare professionals’ attentiveness and stress levels influenced the participants’ sense of safety. The participants understood if the healthcare professionals were mindful of their work or distracted by their next task: “*I perceive if they are mentally present. They make a thorough assessment and are not stressed because the next patient waits for half an hour. You can experience that they have little time for you*” (ID07)*.* The participants reported that when personnel were stressed, they felt less safe, and their feelings of anxiety increased. The response time and availability of healthcare professionals when patients called for assistance affected if they felt safe: “*You feel an uneasiness. You wonder, do I have their attention? Will I get what I need? Should I receive any medication? You need to reassure yourself*” (ID08)*.* Patients perceived that healthcare personnel were less friendly to each other and patients if the atmosphere in the work unit was stressful: “*In some units, they had problems. Patients had to stay in the corridor. Staff were frustrated, and patients were frustrated since staff lacked the time to care for each patient. Often, staff would yell at patients and each other. You feel that without friendliness, they may not do a good job*” (ID02). However, the participants in this study understood that healthcare professionals had stressful working conditions involving prioritizing patients with more severe needs.

#### Subtheme 1b: Emphasizing competence in an organized frame

The participants highlighted structural factors promoting the feeling of being in a well-organized and predictable surgical pathway. Whether an organized care pathway program or not, the patients emphasized the importance of a prepared, integrated, and coordinated pathway: “*I felt that everything was well-organized.* S*omeone had planned the pathway, and now it was my turn. Everyone did the task they were expected to do and let the next do what they were meant to do. I felt very safe”* (ID02). Fragmented healthcare services, organized in silos without communication lines, decreased the participants’ experience of their pathway as coherent. Structures facilitating communication and information flow throughout the surgical path were emphasized to ensure continuity and follow-up. The participants appreciated perceiving that procedures were established and followed. The participants proposed using flowcharts and implementing system barriers, such as second opinions or checklists, to reduce the number of errors in diagnosis and treatment.

The study participants emphasized the managers’ role in proactively promoting predictable pathways and a work environment promoting patient and personnel safety. The patients wanted managers to focus on patient safety and interprofessional risk discussions. The participants perceived that the work environment influenced their sense of safety and appreciated that managers motivated their employees: “*They talk together and have fun together, and you can perceive that they feel safe. It appears they have good managers who take care of them and ensure they can care for the patients best*” (ID06). In addition, the participants were worried about the physicians’ working conditions and their impact on patient safety: “*The physicians are stressed; they work too much and have too many factors to consider. In addition, all their administrative tasks. They are not administrators; they are doctors*” (ID01). They suggested managers should work on regulations for working time, adequate staffing, and physicians’ administrative burden to enable personalized care.

The participants underscored competence and experience as necessary to feel safe. They encountered experienced and inexperienced healthcare professionals. The participants fully understood that the inexperienced professionals had to build competence; however, they highlighted the importance of training programs and the risk of many students and inexperienced personnel in one department. Many participants experienced risk and adverse events because of their experiences with students without supervisors. They perceived that students worked too independently: “*It happens that you feel that the student is too close, too close to the situation, and the supervisor is absent*” (ID08). In other settings, participants felt that the training situation led to adverse events due to the focus on supervision and not on proper treatment. The former surgical patients said that predictable surgical competence, skills, and reputation were necessary to feel safe; “*When it comes to medical procedures, a lot can go wrong. That’s why it’s crucial for healthcare professionals to have both the competence and experience to know exactly what they are doing. I often find it important to know the level of experience a surgeon has before they perform a procedure. There is a significant difference between being operated on by someone who has performed the procedure 2000 times and someone doing it for the second or third time. Ideally, I would like to have the option to choose who performs the surgery* (ID07). The participants valued professional advice when they felt less competent in decision-making. In addition, they emphasized the importance of continuity in their relationship with healthcare professionals. They experienced that continuity facilitated communication and trust and reduced the risk of missing information. The lack of continuity and permanent personnel during holidays reduced their feeling of safety.

### Theme 2: Surgical patients’ involvement: Aspire to be a resource—Not a threat

The participants discussed their potential to engage and use resources throughout their pathway. They had various experiences concerning whether these resources were recognized as essential knowledge in the dialogue and integrated into the medical professional assessment.

#### Subtheme 2a: Patient experiences as untapped resources

The patients shared stories about how they perceived physical signals that made them feel something was wrong: “*I immediately said to my wife that something was wrong. I could feel it; the arm shouldn’t be like this”* (ID10). These subjective perceptions could be patterns of pain or symptoms they could not recognize, and they often included essential knowledge about the patients’ health conditions. However, the participants thought this knowledge was often ignored or not taken seriously. Taking these physical signals into account sooner could reduce treatment delay and influence the prognosis for patients: “*I was not taken seriously when I was certain I had a recurrence of metastasis. It wasn’t until I was admitted to the emergency care department that they realized I had metastasis. You shouldn’t have to be in such dire conditions to be heard. Now, I am a palliative patient*” (ID02).

The participants used information resources throughout their pathways to increase knowledge about their health condition, diagnosis, and treatment. This knowledge empowered them to ask healthcare professionals questions and contribute to an effective and correct diagnosis and treatment; they did not aim to challenge their competence. The patients felt that they valued multidisciplinary approaches more than the healthcare professionals did. They appreciated if healthcare professionals listened to and evaluated these possibilities when health conditions became challenging. “*A multidisciplinary approach can make an essential difference. It's about trust, judgment, and communication where I don't have to repeat myself and can trust that healthcare professionals communicate with each other. It becomes an effective way of working; they get a more holistic perspective and can intervene more quickly. This would have spared me a lot of suffering*” (ID14). Additionally, the participants sought control over the contact and appointments in their care pathways. They experienced that this control was necessary for ensuring the follow-up: “*If I hadn`t checked my appointments, I wouldn`t have gotten any follow-up and would have fallen out of the system because I`m not in a standardized care pathway*” (ID08). In addition, the participants said that their caregivers were essential for demanding assessments or admission when they felt they were not heard: “*If it hadn’t been for my friend demanding that I call again, I wouldn’t have called, and then I wouldn’t be sitting here today*” (ID13).

The participants pointed to how using their resources and knowledge would have been more effective than making patients feel unsafe and ignoring their symptoms, potentially resulting in malpractice: “*I have had CRPS [Complex Regional Pain Syndrome] since my first surgical procedure, followed by many, and the conclusion from the specialist is that this condition is getting even worse after every surgical treatment without taking this syndrome into account*” (ID09). The participants thought that integrating their experiences and healthcare professionals’ medical knowledge could lead to better diagnosis and injury prevention and reduce adverse events’ impact. They wanted to provide resources to facilitate effective coproduction, aiming for optimal outcomes: “*I don't seek healthcare services to tell a story to receive sympathy or empathy; I want help*” (ID05).

The participants suggested that their experiences and those of their caregivers should systematically be obtained to improve the quality of care in the surgical pathway: *“Quality management personnel should assess the patients’ experiences as a measure of quality, not only the quality of the medical treatment*” (ID01).

#### Subtheme 2b: Authoritarian hierarchy as a barrier to interaction

The participants identified challenges limiting their involvement in their healthcare journey. They perceived the interpersonal dynamic between patients and healthcare professionals as unequal, and some perceived that they were expected to be grateful for receiving healthcare services. This asymmetrical power balance limited the participants’ ability to share their perspectives and engage in their pathways. In addition, the healthcare providers sometimes spoke rudely to the participants and in an authoritarian tone. The participants said that the healthcare providers often trivialized their concerns, ignoring them or speaking over their heads: “*They said, ‘This is normal, completely normal. We’ve done this before; you had the same pain after your last operation.’ But that wasn’t true*” (ID11). Some participants also felt that their opinions were dismissed, and their concerns were attributed to psychological causes: “*I had a major internal bleeding, but they didn’t listen to my pain. They thought my PTSD (posttraumatic stress disorder) was causing me to have a low pain threshold*” (ID12).

Patients perceived the teamwork climate both in the wards and the operating room. A teamwork climate characterized by collaboration, cohesion, and respectful communication made the participants feel safer: “*I can sense the atmosphere and mood in the team. I can sense the cohesion and whether they know each other*” (ID03). They felt safe when they sensed continuity, relationships, and a good working flow in the team. In addition, they identified hierarchical relationships between healthcare professionals in the surgical pathway, especially between physicians and nurses, and evaluated this dynamic as potentially threatening patient safety. The participants said that nurses were less likely to express their observations and suggestions when the teamwork climate was tense and hierarchical. This affected the patients’ sense of safety because the nurses could fail to disclose important information about risk and potential harm: “*When the personnel closest to the patients cannot report what is observed without fear or without getting reprimand, it`s a bad culture in the department*” (ID04).

The participants occasionally sensed power struggles and prestige battles between colleagues within a surgical field. Because of these struggles, they felt that they could not ask for a surgeon they knew or one with particular skills. However, the participants experienced some specialist groups as internally cohesive and closely knitted. They sensed that these groups did not want to refer patients to other specialists or hospitals. They felt these cultures were less interested in their involvement: “*I asked if they could send me to a specialist hospital, but they wouldn’t. They wanted to try to fix it themselves and guaranteed it would be successful or, at the very least, not make things worse. The result was twice as bad*” (ID05). The participants also felt that healthcare professionals kept patients’ involvement at a distance. They suggested this could be because of arrogance and prestige, and underscored that conferring with others in the same or other specialties is a professional strength, not a lack of professional competence: “*The excellent professionals are listening and have two main skills. The first skill is that they can refer to more specialized personnel, and the other skill is that they can cooperate with personnel in another specialty to find a solution*” (ID05). The patients’ involvement was not to threaten the authorities; they wanted a safe pathway guided by their experiences and healthcare professionals’ personalized medical evaluations.

### Theme 3: Time to cultivate a culture that fosters improvements and reconciliation

The participants highlighted a culture of openness and learning after adverse events as essential dimensions of patient safety culture. They regarded acknowledging errors and injuries as a prerequisite for improving healthcare services and facilitating the patients’ reconciliation process after an adverse event.

#### Subtheme 3a: Openness for acknowledgment: a starting point for learning

The former surgical patients emphasized the value of openness after adverse events for patients’ and healthcare professionals’ health and well-being. They connected healthcare professionals’ well-being and safety with patients’ safety. The participants considered that healthcare professionals would perform healthcare services with increased quality in a culture of openness without fear of punishment, judgment, or other work-related consequences: “*You must create a climate where you know that errors may occur and that things may not always go as planned. You don’t have to be scared after committing an error*” (ID03).

Participants perceived that surgical units covering up errors affected their sense of safety. Furthermore, they believed that a culture of infallibility was detrimental to healthcare professionals. Some participants had experienced situations where errors were concealed, liability was denied, and healthcare professionals took a distance from involved parts after adverse events: “*After my surgery, I perceived that they were hiding something. The healthcare system has tried to disprove and trivialize my injury*” (ID10). They related this behavior to professional pride: *“Pride. They have challenges in acknowledging errors and I think they fear a professional crack if they admit an injury”* (ID05). Some participants also encountered a closed professional community when events occurred and perceived excuses such as complex conditions, underlying psychological problems, or expected risks that the patients had consented to: “*I am sitting here alone. The others ensure that they and their community have no fault or blame. Protecting their community is a threat to society. When an error occurs, healthcare professionals must persevere*” (ID13).

The participants underscored that they did not expect healthcare to be error-free. However, they highlighted the need for acknowledging when one occurs. They wanted healthcare professionals and the system to own their errors and report them. Openness in reporting included documenting errors and injuries in the patients’ journals and the reporting systems. One participant noted: “*The physician was told to revise the journal, and they did not report the event as an error. The manager claimed to have been talking to me. That’s a lie. That is to cover the truth because that unit wants to be the best in this country*” (ID05). The participants said that fact-based documentation written in real-time could improve the patients’ quality of care.

The participants understood that the admission and reporting of errors could prevent the same errors from happening again. The participants affected by an adverse event valued organizational efforts to learn from their negative experiences: “*To err is human, but you must strive to learn from it and improve to prevent the same error from affecting the next patient*” (ID07). To improve the culture of openness, the participants suggested that surgical units should have an open dialogue about risk and events and be aware of and work proactively on their culture.

#### Subtheme 3b: The importance of apologizing and following up after injuries

The participants emphasized the importance of receiving an apology after an error: “*It would have helped me if he talked with me and apologized for what happened and explained how it could have happened*” (ID02). Many had difficulties moving forward due to the lack of an apology for an injury they had experienced. Instead of receiving an apology, they had to prove what had happened to them and seek an explanation. Their need for detailed information about how the adverse events occurred and unfolded was essential for their ability to move on after the event. They described that this process concerned reconciliation with their new health condition and the consequences for their physical, psychological, social, economic, and working life. A lack of concrete information was a barrier to reconciliation. The combination of lacking acknowledgment and feeling mistrusted made the lives of some participants almost unlivable. Common experiences among the participants who had experienced a severe adverse event were reduced quality of life and a psychologically challenging fight for adequate follow-up. Additionally, some felt that healthcare professionals attributed their situation to a psychological issue. Thus, they felt manipulated to think they had imagined or misinterpreted the adverse event.

Some thought that the lack of acknowledgment could be due to the threat of an increased workload: “*I think they are afraid of opening a glass they can’t get closed again. It’s a stressful world. I think humans are pressed in various ways, and no one wants more work*” (ID14). But acknowledging the adverse event and providing information could have reduced secondary complications and further errors for several of the participants: “*If my concerns had been taken seriously from the beginning, and the healthcare professionals had worked with it directly and assessed the whole situation, I think I could have avoided so much burden. I’m not sure I would be able to run again, but we could have avoided so many secondary complications, pain, and frustrations in my everyday life*” (ID14). Another participant experienced the positive impact of acknowledgment after an error: “*I have never complained about a failed procedure. I had pain in a foot after surgery, and the pain decreased because of the safety and the close follow-up*” (ID01).

The participants who experienced an adverse event resulting in an injury highlighted the need for follow-up initiated by the involved healthcare professionals. They valued person-centered, interprofessional follow-up and pointed to the challenges of being placed in a queue for further treatment, even though errors in healthcare delivery had put them there. Another perceived barrier to receiving relevant follow-up was the lack of shared information between the hospitals’ physicians and physiotherapists in primary healthcare. Insufficient journal notes concerning adverse events could limit the accuracy of rehabilitations and increase secondary problems. When recognition and information were absent, the patients experienced that they searched and received support from other specialties or instances. Practical and psychological support was necessary for embarking on the recovery period. The participants also preferred family-centered care due to the injury’s influence on the families’ lives: “*All of mine has got a new person in their family, and all of them are suffering. I can`t engage in life as I di*d” (ID10).

The reconciliation and recovery period after a patient’s injury could be experienced as a prolonged fight for legal protection. Economic compensation was not as important for these patients as acknowledging the event. An acknowledgment was legal evidence of the adverse event’s physical consequences, for which the patient’s psychological state could not be attributed. These participants highlighted that acknowledgment, documentation, reporting, and information could have spared them from the juridical fight and allowed them to focus on their physical and psychological recovery. “*If they had acknowledged it immediately, everything might have fallen into place much earlier. We could have avoided ending up where we did. It affects not only me but many others as well. It has been exceptionally exhausting*” (ID05). They suggested that a culture of greater openness could reduce psychological distress and suffering from secondary complications, promote reconciliation, and maintain patients’ trust in the healthcare system.

## Discussion

This study provided insight into former surgical patients’ perspectives on patient safety culture in the surgical context. Their experiences enhanced the understanding of processual and structural contributors to patients’ perceived safety. Personalized care and patient involvement in clinical processes, communication openness, and organizational learning after adverse events were highlighted. However, barriers to these safety dimensions were identified. The results aligned with a previous review of patients’ perspectives on patient safety in hospitals [30] and illustrated the complexity of procedural and structural variables affecting outcomes similar to Donabedian’s framework for quality in healthcare [10].

One of the study’s main results was the importance of healthcare professionals’ actively listening to patients’ perceived symptoms and learning from their experiences. This result was consistent with previous reviews of patients’ perspectives on safety, reporting that patients could identify safety issues and risks [20, 30]. A key point from these reviews supported by this study was that engaging patients in risk recognition could improve patient safety, identify incidents, and prevent injuries by actively listening to patients and caregivers [20, 30]. A previous study reported that the risk of diagnostic errors could be reduced through respectful interaction between physicians and patients, including recognizing patients’ knowledge and their stated symptoms and physicians’ communication skills [31]. The participants in this study emphasized respectful dialogue and personalized care for them to feel safe in their meetings with healthcare professionals. A previous review on communication and patient-centered care in nurse-patient interactions highlighted respect and meeting patients’ needs as fundamental values [32]. For patients to perceive healthcare personnel as legitimate, they must take patients seriously, listening to them, and acknowledging their experiences, which can empower patients to engage more actively [33, 34]. Patients can be empowered by feeling more autonomous and emphasizing their knowledge [35]. Simultaneously, the positive effects of empowering patients are reciprocally connected to the individual patient’s health literacy [35].

The participants in this study had resources and searched for information themselves as former patients; however, they highlighted the need for thorough and precise information from healthcare professionals to feel safe. Knowledge and control are essential dimensions of health literacy and behavior change techniques for increased empowerment [33, 34]. Finding a balance between empowerment and health literacy for vulnerable patients in the surgical context is challenging for healthcare professionals [35]. Communication interactions that inform, educate, and enable patients’ autonomy could contribute to this balance and provide constructive benefits regarding patient knowledge and engagement [36]. An essential result of this study was that patients respect physicians’ complex decision-making and their heavy workload. The patients did not want to increase this workload; they just wanted to contribute their knowledge and experiences to improve diagnosis and treatment. Patient engagement should be included in educational curricula and clinical settings, according to evidence demonstrating that integrating patient experiences in medical evaluations could be efficient, reduce costs, and increase healthcare outcomes [5, 31, 32, 34, 35].

This study pointed to hierarchical relationships as potential cultural barriers to engaging patients in their pathways. Villar [30] demonstrated that poor communication was perceived as contributing to adverse events and resulting in a disrespectful relationship between healthcare professionals and patients. Ignoring patient knowledge was problematic behavior [19, 31]. Some participants in this study experienced healthcare professionals ignoring their concerns, resulting in severe consequential errors, secondary issues, and a reduced quality of life. These stories were similar to a previous review on patients’ and families’ perceptions of patient safety culture, which reported that patients experienced being ignored [20]. This review noted that patients perceived barriers to speaking out when their intuitive feelings indicated something was wrong. If they raised their concerns, they often felt that healthcare professionals were reluctant to recognize their experiences [20]. The participants had similar experiences and felt that authoritarian attitudes acted as a barrier to being acknowledged. Some of the patients had experienced healthcare professionals trivializing and exhibiting incivility when expressing symptoms and concerns. Professional authorities might be changed and challenged by patient engagement as a safety pillar [37, 38]. In contrast, this study and a previous study reported that engaged patients did not aim to challenge professional authorities; they aimed to support decisions [38]. A communicative change from “tell-ask-tell” to “ask-tell-ask” in medical teaching could acknowledge patient knowledge [37]. However, patients still perceive healthcare professionals as the most trustworthy and reliable information source, so the “tell” in the middle should still be emphasized and maintained as a well-established safety pillar [37, 38]. Assessing surgical patients’ situated health literacy [35], empowering them in interactions with patients’ surgical safety checklists [39], and integrating patient knowledge into physicians’ medical evaluations could increase the quality of healthcare processes [2, 3, 5].

The participants in this study connected a poor teamwork climate and incivility among coworkers to hierarchies, incivility toward patients, and decreased feeling of safety. Incivility among healthcare professionals has been demonstrated to be negatively associated with patient safety culture and patient outcomes [40]. The participants said that they felt better communicating with nurses who know their needs. Culture, authority, and hierarchy affect the nurses’ ability to speak out [41]. In a previous study on surgical teams’ perspectives on patient safety culture, physicians also encouraged nurses to speak out about risk and safety issues [42]. However, the traditional hierarchy in the surgical context makes it necessary for managers and formal and informal role models to encourage the team to speak out, thus promoting a better teamwork climate and improving patient safety [41, 42]. This study added that nurses should engage and speak out on behalf of vulnerable patients’ experiences, thus integrating patient and professional knowledge. Making nurses feel more comfortable speaking out could prevent injuries and increase safety [43, 44].

The participants’ experiences after severe adverse events should be critically examined. They experienced a lack of acknowledgment when an injury occurred. A previous study on patients’ perspectives on disclosing adverse events reported a lack of openness to injuries [19]. The WHO’s Global Safety Action Plan 2021–2030 states that disclosing incidents to harmed patients is a strategy for engagement [1]. The lack of acknowledgment that the participants in this study experienced consequently led to missed apologies and the loss of pertinent information. Sattar [18] reviewed the evidence of the disclosure of adverse events and reported that patients and healthcare professionals advocate for disclosure. However, they represented different attitudes and expectations, and the patients highlighted the need for sincere apologies and information [18]. This study supported these findings and added that acknowledgment of adverse events is necessary for a follow-up plan and to prevent secondary consequences. Haagensen [19] also demonstrated that affected patients struggled to prove the links between events and injuries and to receive essential follow-up. Similarly, the participants in this study had to prove that healthcare performance caused their injuries, and they pointed to a lack of documentation in patient journals and hospitals’ reporting systems as additional barriers regarding this burden of proof. This could be because healthcare professionals tend to underestimate the burden of adverse events compared with patients’ evaluations [19]. Moreover, only a minor number of adverse events documented in the patients’ journals are demonstrated to be registered in the reporting system [45, 46].

The participants in this study did not receive disclosure after adverse events. Sattar [18] reported healthcare professionals’ cultural barriers to disclosing adverse events. They expressed challenges in reporting events in a culture of blame, avoiding legal disputes, and communicating disclosure and guidance [18]. In addition, the former surgical patients suggested that the prestige culture of healthcare services could maintain a closed culture, limiting communication openness. The participants’ experiences of missing documentation and evidence indicated a need for improved communication systems and a better culture for disclosing and documenting adverse events. Highlighting system issues after adverse events were reported to increase error reporting and improve patient outcomes; thus, it should be considered for improved quality of care and education [47]. The patients emphasized that “to err is human”; they did not expect a healthcare system without adverse events. However, they preferred that their experiences lead to organizational learning and interventions to prevent other patients from experiencing the same error. This was supported by Sattar [18], who reported that patients want a promise that the hospital and healthcare system have learned and implemented improvements to prevent further errors.

Understaffing and heavy workloads were suggested as barriers to a culture of openness after adverse events. Surgical team members work in stressful environments, and burnout is described as a new pandemic [48, 49]. Experiencing an adverse event further increases moral distress and emotional exhaustion [50], and these predictors of burnout are associated with decreased patient safety and poorer patient outcomes [51]. The participants in this study suggested that healthcare professionals might experience disclosing adverse events as overwhelming, especially if they were already exhausted. However, the patients emphasized that, over time, the disclosure of adverse events could promote well-being for both patients and healthcare professionals. To further improve patient safety, healthcare professionals can approach and use patient knowledge more systematically [52]. Thus, the results of this study can also serve as a crucial reminder of patient involvement and knowledge to increase the patient safety culture. Interventions for acknowledging after events, promoting a culture of openness, providing real-time documentation, and improving organizational learning at the system level could increase patient safety culture and improve patients’ experiences and the challenging reconciliation process. The team should not perceive engagement as threatening their authority or increasing their workload; professional medical education and knowledge are well-established pillars, and patient engagement and error acknowledgment could improve the effectiveness and robustness of this pillar.

### Strengths and limitations

This study had multiple strengths. First, the themes and patterns of meaning were generated from empirical material from a heterogeneous group of strategically sampled participants [25]. Second, the interviews were primarily driven by the participants’ stories. They shared their experiences openly, thus reducing the risk of moderators’ influence on their perspectives. Third, the iterative and reflective dynamic process in the analysis panel increased the validation of the final themes. This study also had limitations. First, the participants had experienced adverse events, and these experiences affected their shared narratives and what is highlighted in this study. Many of the participants were also grateful for the professional way they were treated as patients. The reality that most surgical procedures and pathways are successful was given less attention in the study. Second, despite the reflections related to the mind map before the interviews and the emotional engagement the participants shared during the interviews, the risk of recall bias could not be eliminated. Third, voluntary participation might have resulted in recruiting participants with resources and the capacity to share their perspectives. However, they could still represent those with fewer resources.

## Conclusion

This study provided insights into patient safety culture and adverse events in the surgical context by highlighting the perspectives of former surgical patients. Patients emphasized personalized care, equal dialogue, and information sharing during their pathways, and their perceptions of team climate and the working atmosphere influenced their sense of safety. However, patients’ resources were often untapped, and their experiences were often ignored. In addition to these processual variables, structural variables, such as well-organized pathways, competence, continuity, and adequate staffing, influenced their perceived risk and safety. Patients often failed to receive an acknowledgment of their severe adverse event, documentation in their medical records, and adequate follow-up. They related this to cultural aspects, such as hierarchy, authoritarian attitudes, and a culture of prestige. The study identified the need to acknowledge patient knowledge as a pillar in patient safety work and its importance for professional medical evaluation. Interventions to foster a psychologically safe culture could increase team performance and promote shared accountability. In addition, promoting a culture of openness in educational and clinical settings could provide organizational learning and facilitate reconciliation for patients affected by adverse events. Finally, patients’ perspectives should be complemented by caregivers’ and managers’ perspectives.

## Supplementary Information

Additional file 1. Mind Map.

Additional file 2. Interview Guide.

## Data Availability

The data is not publicly available to ensure participants’ confidentiality. However, the corresponding author can provide the data upon reasonable request.
