# Supplementary material for: Patient safety culture through the lenses of surgical patients: a qualitative study
Source: BMC Health Serv Res. 2025 Feb 7;25:215. doi: 10.1186/s12913-025-12366-9 (PMC11804094; doi:10.1186/s12913-025-12366-9)
Supplement: Supplementary file 2 — Additional file 2. Interview Guide. [file 12913_2025_12366_MOESM2_ESM.docx]

# Interview guide for former surgical patients

**Introduction: Information about the project and privacy and questions about characteristics: gender, age, the field of surgery, elective or acute surgery, level of education, occupation, and the number of years since their experience as a surgical patient.**

**First, can you tell me about your experiences being cared for as a patient from when you were admitted, during the hospital stay, and until you were back home? (1-admission, 2-hospital stay, 3-discharge)? Possible follow-up questions:**

- Could you describe an episode where you felt particularly well cared for? (How did this influence your experience? What did this do to you? Why do you think you reacted that way or experienced it like that? What made you feel well taken care of?)
- Could you describe an episode where you did not feel well cared for? (How did this influence your experience? What did this do to you? Why do you think you reacted that way or experienced it like that? What made you feel not as well taken care of?)

**What did patient safety mean to you while you were a patient? Possible follow-up questions:**

- How can physicians influence your perception of safety as a patient? How can nurses influence your perception of safety as a patient? What other factors influenced your perception of safety as a patient?

**We often talk about “adverse events” in healthcare in general and specifically in hospitals. These can involve errors or deficiencies in treatment that cause or could have caused harm or additional burden to patients.**

- Did you experience any adverse events, errors, or injuries you would like to share? How did you feel taken care of in that situation? How did the involved physicians and nurses handle the situation? What do you think influenced how they responded? Why do you think the event occurred?
- Did you experience a situation where an error or an event could have caused harm in some way while you were a patient? How did the healthcare professionals handle this situation? Was there anything in that situation that you think influenced the risk? What factors prevented the situation from becoming a patient injury? How did this affect your perceptions of safety?
- How should healthcare professionals ideally respond to and handle adverse events or patient injuries?

In summary, is there anything you want to say that you haven’t had the chance to mention?

**Summarize and clarify**
